# Screening and surveillance for retinopathy of prematurity: A Wilson and Jungner framework approach

**DOI:** 10.7189/jogh.13.03028

**Published:** 2023-05-19

**Authors:** Sam Ebenezer Athikarisamy, Geoffrey C Lam

**Affiliations:** 1 Neonatal Directorate, Child Adolescent Health Service, Perth, Western Australia; 2 School of Medicine, University of Western Australia, Crawley, Western Australia; 3 Department of Ophthalmology, Perth Children’s Hospital, Perth, Western Australia; 4 Centre for Ophthalmology and Visual Science, University of Western Australia, Crawley, Western Australia

**Figure:**
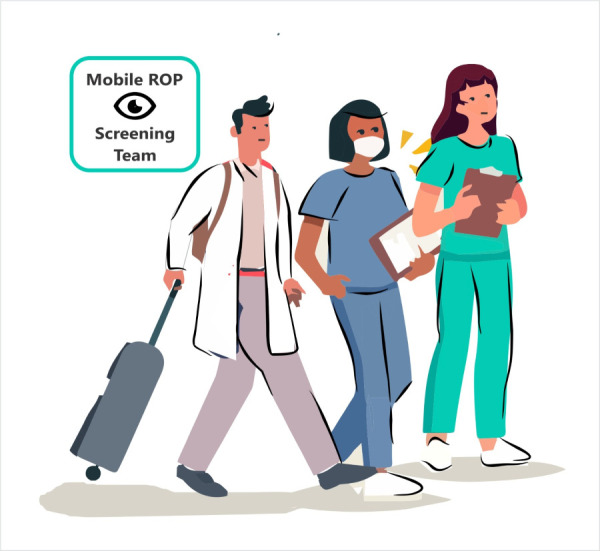
Photo: Imaging staff en route to the neonatal unit for wide-field digital retinal photography screening of ROP. Source: Designed by Dr Aditee N Vaidya, used with permission.

Retinopathy of prematurity (ROP), a potentially blinding disease, has emerged in distinct epidemics, with low birth weight, gestational age, and exposure to oxygen identified as primary risk factors [1]. Understanding its epidemiology has highlighted the need for prevention through public health policies and interventions. In low- to middle-income countries, unmonitored oxygen exposure and lack of ROP screening have emerged as significant risk factors for ROP-related blindness [2]. While evidence-based approaches for oxygen targeting, reducing risk for sepsis, and optimising nutrition serve as primary prevention measures, ROP screening is equally critical in identifying at-risk infants and initiating timely interventions to prevent blindness. Wilson and Jungner's guidelines for disease screening have been a major reference for population-based screening decisions since their publication in 1968 [3]; by applying their “ten principles” to the ROP screening program, we aim to highlight the importance of early detection and treatment of ROP to prevent blindness and lifelong vision impairments in preterm infants.

## APPLICATION OF THE WILSON AND JUNGNER CRITERIA FOR ROP

### The condition should be an important health problem

ROP is a common avoidable cause of childhood blindness, causing an estimated 32 300 cases of blindness and visual impairment globally in 2010, with the highest incidence in middle-income countries with expanding neonatal care coverage [4]. In 2022, Zhang et al. [5], based on cause-specific vision loss data from the Global Health Data Exchange, estimated that ROP caused moderate vision loss in approximately 49 100 cases, severe vision loss in 27 500 cases, and blindness in 25 000 cases in 2019 [5]. The study found that the prevalence of ROP-related blindness varied among 21 Global Burden of Disease regions, with southern sub-Saharan Africa having the highest rate at 44 cases per 100 000 population and East Asia having the lowest rate at 17 cases per 100 000 population [5]. By 2050, estimated global prevalence of moderate, severe vision loss, and blindness caused by ROP is projected to reach 43 600, 23 200, and 31 900 per 100 000 population, respectively [5]. Blindness in childhood increases the likelihood of socioeconomic deprivation, frequent hospitalisations, and early death [6].

### There should be an accepted treatment for patients with recognized disease

Screening guidelines for retinopathy of prematurity (ROP) are available in many countries; they vary across healthcare systems and are typically based on factors such as gestational age, birth weight, and disease pattern [7]. Peripheral retinal cryotherapy was found to be effective in reducing unfavourable outcomes for threshold ROP in the multicenter CRYO ROP trial [8]. Laser photocoagulation is now the preferred method of ablation for ROP, and ETROP study protocols have been used for treatments [9]. Randomised controlled studies, including the “Bevacizumab Eliminates the Angiogenic Threat of Retinopathy of Prematurity” (BEAT-ROP) [10] and the “RAnibizumab Compared With Laser Therapy for the Treatment of INfants BOrn Prematurely With Retinopathy of Prematurity” (RAINBOW) [11], have investigated the effectiveness of anti-vascular endothelial growth factor (VEGF) agents for treatment. Other agents, such as aflibercept, are being investigated [12]. While the use of anti-VEGF agents has increased over the last ten years, the ideal choice of agent and optimal dosing is still being debated, given their unknown long-term effects. In some clinical scenarios, a combination of laser photocoagulation and anti-VEGF therapy may be used [13,14]. Despite variations in screening guidelines across countries and healthcare systems, the treatment principles for ROP remain similar.

### Facilities for diagnosis and treatment should be available

In high-income countries, ROP screening and treatment services have been well integrated within neonatal intensive care and their follow-up programme. However, in low- to middle-income countries, the lack of ophthalmologists and inadequate healthcare infrastructure has led to patchy coverage for screening and treatment. ROP screening using wide-field digital retinal photography by non-ophthalmologists is an alternative to screening by ophthalmologists [15]. The expansion of the healthcare workforce for the diagnosis and treatment of ROP is needed, and in the future, an artificial intelligence (AI)-integrated wide-field digital retinal photography system could serve a larger scale of at-risk infants [16].

### There should be a recognizable latent or early symptomatic phase

While the progression of ROP may vary among cases, certain stages and patterns tend to follow a predictable sequence, which is why timely retinal examinations are crucial in identifying and treating ROP before it becomes more severe, potentially leading to blindness [14]. Timely treatment is important to minimise the risk of retinal detachment and should be initiated as soon as possible, at least within 72 hours of identifying treatment-warranted disease [14].

### There should be a suitable test or examination

The preferred method for retinal examinations is the binocular indirect ophthalmoscope (BIO), considered the standard for ROP screening [14]. ROP screening should be scheduled based on the infant's gestational age and disease severity, and all primary care providers should be aware of the schedule. Country-specific guidelines are available to guide clinicians in the timing of initial screening and follow-up. In low- to middle-income countries and certain clinical settings in high-income countries, telemedicine systems using wide-angle retinal images are being increasingly used for ROP screening or as a complement to BIO. AI is being explored as a potential tool for ROP screening but is still in early phases of development and testing [17].

The International Classification of Retinopathy of Prematurity (ICROP) is a classification system that is globally utilised to describe and classify stages of ROP based on the location and extent of retinal abnormalities and to ensure uniformity in diagnosis and treatment recommendations. It was first published in 1984 and later expanded in 1987 as a consensus statement [18,19]. The 2005 revised ICROP classification introduced the concept of aggressive, posterior ROP in the tiniest babies, “an intermediate level of plus disease (preplus), and a practical clinical tool for estimating the extent of zone I” [20]. The ICROP3 (2021) introduces several significant updates to its classification metrics, including more detailed descriptions of the “posterior zone II and notch, the subcategorization of stage 5, and a recognition that there is a continuous spectrum of vascular abnormality that ranges from normal to plus disease” [21]. The term “aggressive-posterior ROP” has been updated to “aggressive ROP”, and “regression and reactivation” of ROP and long-term sequelae are detailed in this update [21].

### The test should be acceptable to the population

While the nature of ROP examination may be uncomfortable for premature infants [22] and their parents, the potential consequences of not undertaking the screening could be more severe, including permanent vision impairment or blindness. By providing information about the potential benefits of early detection and treatment of ROP, caregivers can make informed decisions and better understand the importance of the screening program. Ultimately, the benefits of ROP screening far outweigh the associated temporary discomfort, and education can help ensure that the screening and surveillance program is acceptable to the population.

### The natural history of the condition, including development from latent to declared disease, should be adequately understood

ROP is more common in premature infants with lower birth weight and gestational age. Overall, all stages of ROP can undergo complete spontaneous resolution in 90% of infants, but the incidence and severity of cicatricial sequelae increase with decreasing birthweight and gestational age [23,24]. The CRYO-ROP study provided unique and well-documented information on the natural history of eyes with “advanced acute retinopathy of prematurity” that received no treatment. As severe ROP is now universally treated, this cohort is unlikely to be replicated [25]. Prior to this study, the investigators expected that approximately 50% of untreated eyes would have unfavourable outcomes, and this expectation was validated with 53% of untreated eyes showing unfavourable fundus appearance [8]. For most infants with Zone 1 and Zone II ROP, signs of involution appeared around a mean postmenstrual age of 38-39 weeks and before 45 weeks in most infants (95th percentile). In contrast, the involution process for Zone III disease was significantly prolonged (week 34.0-50.6, 5%-95%). However, the outcome in zone 3 was favourable more than 99% of the time [26].

### There should be an agreed-upon policy on whom to treat as a patient

The Early Treatment of Retinopathy of Prematurity Randomized Trial (ETROP) established new guidelines for the treatment of ROP, defining “type 1 ROP as aggressive and requiring treatment and type 2 ROP as less aggressive” [9]. Most units around the world follow these guidelines, recommending treatment for type 1 ROP, which is “defined as zone I, any stage ROP with plus disease, zone I, stage 3 ROP without plus disease, or zone II, stage 2 or 3 ROP with plus disease”. The ETROP guidelines recommend a wait-and-observe approach for type 2 ROP, as it is generally less aggressive and may not require immediate treatment. However, close monitoring and timely intervention may be necessary if it progresses to type 1 ROP [9].

### The cost of case-finding (including a diagnosis and treatment of patients diagnosed) should be economically balanced in relation to possible expenditure on medical care as a whole

The main objective of ROP screening is to preserve the visual health of preterm infants by identifying at-risk infants and initiating appropriate interventions. While screening for retinopathy of prematurity (ROP) may involve costs, it can be considered cost-effective when compared to the potential costs of blindness to the health care system. A systematic review of 15 studies examined the lifetime costs of ROP and its associated treatment and follow-up costs [27]. The review focused on 13 studies that evaluated costs related to treatment or follow-up of ROP. The results of the review showed that the costs of screening and treatment for ROP were relatively small compared to the costs associated with blindness caused by the disease.

### Case-finding should be a continuous process and not a “once and for all” project

ROP screening is an ongoing process, and specific criteria exist for completing the screening process. The decision to stop retinal examinations for ROP should be determined based on the infants’ age and the findings of ophthalmoscopic examination of the retina.

Screening may be terminated when full retinal vascularisation is achieved and “when zone III retinal vascularization is attained without any previous zone I or II ROP” [14]. In the absence of type 1 or worse ROP, examinations may also be terminated at a postmenstrual age (PMA) of 45 weeks. If anti-VEGF agents are administered, infants need to be closely monitored for signs of disease reactivation, which can occur up to 69 weeks PMA [28]. Care must also be taken to “ensure that no abnormal vascular tissue is present in zone II or III” that is capable of reactivation and progression [14].

## DISCUSSION

**Figure 1:**
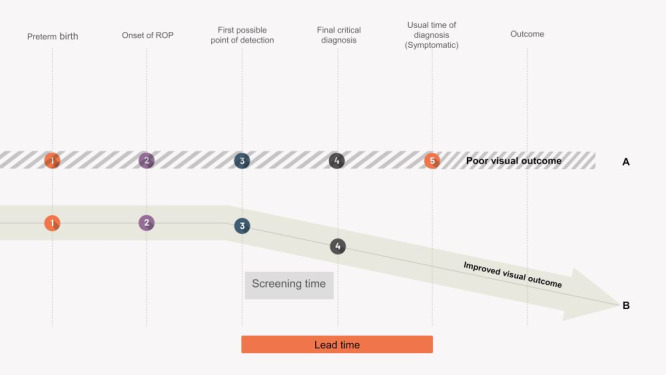
Schematic representation of the outcomes for infants with ROP with and without screening. Pathway A represents the outcome of an unscreened infant with Type 1 ROP. Pathway B represents the outcome of an infant who completed screening for ROP.

In summary, the ROP screening program meets all “ten principles” of screening laid out by Wilson and Jungner [3]. Fulfilling these ten criteria in a satisfactory manner, regular screening has the main advantage of a substantial gain in “lead time”, which is defined as the time that elapses between the point at which changes can be detected and the point at which the disease would have expressed itself symptomatically had it not been detected by screening. This is very true for ROP, as infants or parents may not notice any symptoms until the retina is detached (**Figure 1**). To enhance the effectiveness of screening programs, some countries have gone beyond the basic principles and introduced additional criteria. Sweden, for example, has incorporated five supplementary criteria that pertain to the program's organization, resource needs, feasibility, cost-effectiveness, and follow-up [29]. Effective management of retinopathy of prematurity (ROP) involves not only initial screening but also ongoing “surveillance” through multiple examinations over time to ensure timely detection and treatment of ROP, as well as monitoring after treatment to assess for recurrence or complications. The term “surveillance” highlights the need for continued attention and resources to address the ongoing health needs of preterm infants and emphasizes the importance of continued funding and support for ROP programs.

The rationale for screening for ROP both in terms of human and financial perspectives seems unquestionable. Further understanding of the epidemiology and etiologies of ROP is needed to develop strategies to prevent and reduce burden. Primary prevention strategies include improving oxygen practices, while ROP screening plays a crucial role in identifying at-risk infants and initiating appropriate interventions to preserve the visual health of preterm infants.
